# Decoronation as a Surgical Technique for Managing Ankylosed Permanent Anterior Teeth in Growing Patients: A Systematic Review

**DOI:** 10.3390/healthcare14131811

**Published:** 2026-06-23

**Authors:** Gwendelyn Bulosan Laurencio, Tawfiq Hijazi Alsadi, Agustina Muñoz Rodríguez, Kais Hijazi Muwaquet, Susana Muwaquet Rodriguez

**Affiliations:** 1 Department of Dentistry, Faculty of Medicine and Health Science, Catholic University of Valencia, 46001 Valencia, Spain; 2 Department of Orthodontics, Faculty of Medicine and Health Science, Catholic University of Valencia, 46001 Valencia, Spain; 3 Department of Preventive Dentistry and Epidemiology, Faculty of Medicine and Health Science, Catholic University of Valencia, 46001 Valencia, Spain; 4 Department of Restorative Dentistry and Endodontics, Faculty of Medicine and Health Science, Catholic University of Valencia, 46001 Valencia, Spain

**Keywords:** alveolar bone preservation, decoronation, dental ankylosis, growing patients, infraocclusion, implant rehabilitation

## Abstract

**Background:** Dental ankylosis (DA) in growing patients leads to progressive infraocclusion and alveolar ridge deformities, compromising future implant rehabilitation. Decoronation has been proposed as a biologically driven alternative to extraction for preserving alveolar bone during growth. **Objective:** We aimed to evaluate the clinical outcomes of decoronation—alveolar ridge preservation, infraocclusion progression, implant site development, and the influence of treatment timing—in growing patients with ankylosed permanent anterior teeth. **Methods:** This systematic review was conducted in accordance with PRISMA 2020 guidelines. A comprehensive search of MEDLINE (EBSCO), EMBASE, Scopus, and Web of Science was performed (January 2006–May 2026), supplemented by grey literature screening. Eligible studies included clinical investigations reporting outcomes of decoronation in patients ≤18 years. Risk of bias was assessed using the Newcastle–Ottawa Scale (NOS) and Joanna Briggs Institute (JBI) checklist. Certainty of evidence was evaluated using the GRADE framework. Lastly, an inter-rater agreement was quantified using Cohen’s kappa coefficient. **Results:** Five studies (two retrospective cohorts and three case series) comprising 140 decoronated teeth with follow-up periods ranging from 1 to 30 years were included. A total of 78 records were identified across four databases; five studies met the eligibility criteria after duplicate removal and screening. Inter-rater agreement at the full-text eligibility stage was good (κ = 0.70). The overall risk of bias was low to moderate, and the certainty of evidence was rated as low using the GRADE framework. Vertical alveolar bone preservation or gain was consistently observed, particularly when decoronation was performed during the prepubertal or pubertal growth phases. The largest cohort (*n* = 103) reported substantial vertical bone gain when intervention occurred at a mean age of 13.0 years in girls and 14.6 years in boys. Infraocclusion stabilisation or improvement was reported across all studies. In contrast, horizontal ridge reduction persisted, with the only quantitative study reporting a mean bucco-palatal loss of 1.67 ± 1.12 mm (*p* = 0.004). No included study directly assessed implant placement outcomes. Overall, the certainty of evidence was low due to observational study designs, heterogeneity in outcome assessment, and absence of controlled comparators. **Conclusions:** Decoronation appears to be a promising strategy for preserving vertical alveolar bone and stabilising infraocclusion in growing patients with ankylosed teeth, particularly when performed before or during the pubertal growth phase. Evidence showed considerable bone height preservation, though horizontal ridge reduction persisted across cases. However, the certainty of evidence remains low because available studies are observational, heterogeneous, and lack direct extraction comparators. Therefore, high-quality prospective studies with standardised outcome measures and controlled comparisons are required to establish definitive clinical protocols. Participants underwent decoronation during childhood or adolescence (≤18 years); reported follow-up periods of up to 30 years reflect monitoring that extended into adulthood. **Clinical significance:** For clinical decision-making, decoronation should be considered once ankylosis with progressive infraocclusion is confirmed during active growth, ideally before the pubertal spurt; the decision should be guided by growth stage rather than chronological age, and clinicians should anticipate likely horizontal ridge reduction by planning for possible augmentation at implant placement and coordinating multidisciplinary follow-up until skeletal maturity.

## 1. Introduction

Dental ankylosis (DA) is a pathological condition in which the root surface fuses directly with the surrounding alveolar bone following destruction of the periodontal ligament (PDL) [1]. The absence of the PDL results in replacement resorption, a sterile, progressive process in which the dental hard tissues are gradually resorbed and replaced by newly formed bone [2,3]. Because ankylosed teeth cannot erupt in synchrony with adjacent dentition, the affected alveolar bone fails to develop vertically, and the tooth progressively falls below the occlusal plane—a phenomenon known as infraocclusion (IO). Clinically, ankylosis most often affects previously erupted permanent incisors that subsequently undergo progressive IO, although partially erupted, retained, or impacted teeth may also be affected. The severity of infraposition is commonly graded as mild, moderate, or severe. Although traumatic injury is the predominant aetiology, ankylosis may also arise from idiopathic causes, periapical pathology, or a hereditary predisposition. Diagnosis is based on the absence of physiological mobility, a characteristic high-pitched metallic percussion sound, and radiographic loss of the PDL space with evidence of replacement resorption [4,5].

Ankylosis is a multifactorial condition commonly associated with traumatic dental injuries, particularly avulsion and intrusive luxation [6,7]. This is particularly relevant after avulsion injuries with delayed reimplantation, where extensive PDL damage can initiate replacement resorption [1,7]. Dental trauma in this population, therefore, carries significant long-term consequences: progressive IO, arrested alveolar growth, ridge defects, and compromised aesthetic and functional outcomes that may ultimately jeopardise future implant rehabilitation [8,9].

Conventionally, extraction has been the standard intervention for DA. Extraction nonetheless remains a valid and appropriate option in selected cases, for example once skeletal maturity is reached or where the residual root is severely infected. However, when performed during active growth it may accelerate both vertical and horizontal bone loss due to the premature removal of the tooth, where the root acts as a scaffold to maintain the alveolar ridges [10]. Since implant placement in these individuals is delayed, the resulting ridge defects usually require bone augmentation, which increases morbidity and treatment complexity [9].

Decoronation, first described by Malmgren and co-workers in 1984, was proposed as a biologically conservative alternative [11]. The technique involves removal of the crown and reduction in the coronal root portion below the alveolar crest, with the root retained in situ to undergo gradual replacement resorption. The biological rationale rests on two mechanisms: first, a new periosteum forms over the decoronated root, which allows the continued vertical bone development as the root is replaced by bone; second, the interdental fibres severed during decoronation reorganise between adjacent teeth, and the continued eruption of the neighbouring teeth drives marginal bone apposition via the dentoperiosteal fibre complex [11,12]. Collectively, this mechanism transforms a potentially detrimental clinical condition into a controlled remodelling environment where the ridge develops along with the adjacent erupting dentition [7].

Current International Association of Dental Traumatology (IADT) guidelines recognise decoronation as a valid treatment option for ankylosed permanent teeth in growing patients, with particular relevance to anterior teeth [7]. Growth stages in this review are defined as follows: the prepubertal stage refers to the active skeletal growth before the onset of puberty (Cervical Vertebral Maturation [CVM] stages CS1–CS2, generally below 10–12 years in girls and 11–13 years in boys); the pubertal stage corresponds to the period of peak skeletal growth velocity (CVM stages CS3–CS4); and the post-pubertal stage, or skeletal maturity, follows completion of active growth (CVM stages CS5–CS6), after which vertical alveolar bone apposition following decoronation is no longer expected. Growth stage, rather than chronological age, is therefore the critical determinant of treatment outcome.

However, only a limited number of clinical studies meeting robust eligibility criteria are available, none directly compare decoronation with extraction in a controlled study design, and none report implant placement as a primary outcome [13]. Heterogeneity in outcome measures—including bone height, ridge width, and IO progression—further complicates the interpretation of existing results, and the optimal timing of intervention in relation to growth stage remains undefined. Previous systematic reviews have only partly addressed these gaps: the earliest synthesis applied no minimum follow-up threshold and excluded cohort studies, while the most recent review evaluated vertical bone change alone. The present review therefore offers a broader, design-matched synthesis that additionally examines horizontal ridge change, infraocclusion progression, growth stage as a modifying factor, and implications for future implant placement, interpreting the findings against indirect post-extraction resorption data.

The present systematic review was therefore undertaken to synthesise the current clinical evidence on decoronation as a ridge-preservation strategy in this population with ankylosed permanent teeth, to identify the influence of intervention timing on outcomes, and to highlight research gaps relevant to future implant rehabilitation.

## 2. Research Question

What are the clinical outcomes of decoronation in growing patients with ankylosed permanent anterior teeth in terms of vertical and horizontal alveolar ridge changes, infraocclusion progression, and the influence of treatment timing relative to skeletal growth stage?

## 3. Objectives

To systematically evaluate the effect of decoronation on alveolar ridge preservation in growing patients with ankylosed permanent anterior teeth.

More specifically, this review aimed to: assess the effect of decoronation on IO progression; evaluate its impact on future implant site conditions; analyse aesthetic outcomes following treatment; determine the influence of timing and growth stage on outcomes; and, where available, compare outcomes with extraction or interpret them in relation to indirect evidence from post-extraction ridge-resorption studies.

## 4. Materials and Methods

### 4.1. Study Design

This systematic review was conducted in accordance with the *Preferred Reporting Items for Systematic Reviews and Meta-Analyses* (PRISMA) 2020 guidelines [14] to ensure methodological transparency and reproducibility. Adherence to PRISMA reporting items is detailed in a Supplementary Table S1. The protocol was registered in the Open Science Framework (OSF) [https://osf.io/kbn8j, accessed on 19 May 2026]. The following PICO question guided the review: ‘*What are the clinical outcomes of decoronation in growing patients with ankylosed permanent anterior teeth?*’.

Population (P): Growing patients (prepubertal, pubertal, or adolescent) with ankylosed permanent anterior teeth undergoing replacement resorption.

Intervention (I): Decoronation.

Comparison (C): Extraction.

Outcome (O): Vertical and horizontal alveolar ridge changes, infraocclusion progression, and factors influencing treatment outcomes (e.g., timing relative to growth stage).

### 4.2. Eligibility Criteria

Growth stages were interpreted according to the definitions provided in the Introduction, using prepubertal, pubertal, and post-pubertal/skeletal maturity categories based on Cervical Vertebral Maturation stages. The eligibility criteria are summarised in Table 1 and Table 2.

### 4.3. Search Strategy and Study Selection

The search was conducted in November 2025 and updated in May 2026 across four databases: MEDLINE (via EBSCO), EMBASE, Scopus, and Web of Science. Studies published from January 2006 to May 2026 were considered eligible. Medical Subject Headings (MeSH) and free-text keywords were combined using Boolean operators (AND, OR). The database-specific search strategies are summarised in Table 3. Grey literature was additionally screened through Google Scholar, and reference lists of included studies were manually screened.

In addition, two reviewers (G.B.L. and S.M.R.) independently screened records at the title, abstract, and full-text stages. Discrepancies were resolved by consensus discussion. Inter-rater agreement across 21 studies at the full-text screening stage was assessed and quantified using Cohen’s kappa coefficient (κ), calculated from the 2 × 2 contingency table, with values interpreted according to Landis and Koch (κ < 0.20 poor; 0.21–0.40 fair; 0.41–0.60 moderate; 0.61–0.80 good or substantial; 0.81–1.00 almost perfect). A κ ≥ 0.61 was considered acceptable for proceeding with study selection.

### 4.4. Data Extraction and Synthesis

The data were independently extracted by two reviewers (G.B.L. and S.M.R.) using a piloted extraction form, with discrepancies resolved by consensus. Extracted variables included: author, year, country, study design, sample size, patient age, sex, tooth type, ankylosis diagnosis, trauma history, growth stage, timing of decoronation, follow-up duration, radiographic method, vertical alveolar bone outcome, horizontal ridge-width outcome, infraocclusion outcome, implant or prosthetic outcome, aesthetic outcome, and complications.

The limited number of eligible studies and the lack of homogeneous outcome measurement methods across studies precluded a meta-analysis. As a result, the findings were reported as a narrative review. Quantitative pooling was considered inappropriate owing to the small number of studies (*n* = 5), the diversity of study designs (cohort studies and case series), and the substantial clinical and methodological heterogeneity in outcome definitions, measurement methods, and follow-up intervals. Relaxing the eligibility criteria to increase the number of studies would have introduced reports that do not directly address the review question, thereby compromising internal validity. A structured narrative synthesis was therefore judged to be the most rigorous and transparent approach to the available evidence.

### 4.5. Quality and Risk of Bias Assessment

The methodological quality of the included studies was evaluated using the Newcastle–Ottawa Scale (NOS) for retrospective cohort studies [15] and the Joanna Briggs Institute (JBI) Critical Appraisal Checklist for Case Series [16]. Studies attaining scores 7–9 on the NOS or ≥70% on the JBI checklist were considered to have a low risk of bias. Furthermore, two reviewers (G.B.L. and S.M.R.) performed the appraisal independently, and any discrepancies were resolved by consensus.

The certainty of the body of evidence for each pre-specified outcome was additionally appraised using the Grading of Recommendations, Assessment, Development and Evaluations (GRADE) framework. The full GRADE Summary of Findings table, including domain-by-domain ratings and explanatory footnotes, is provided in Supplementary Table S2.

## 5. Results

### 5.1. Study Selection

**Figure 1:**
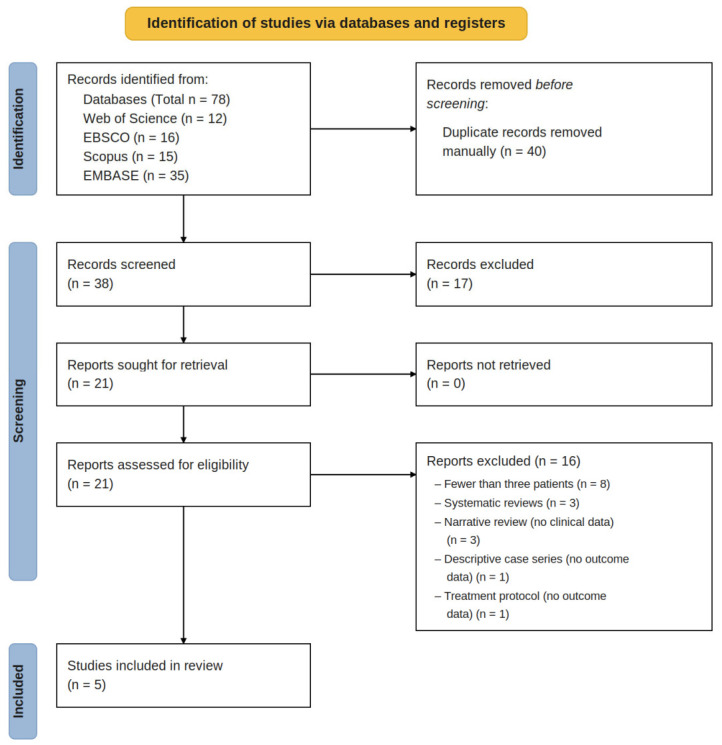
PRISMA 2020 flow diagram outlining the study selection process.

A total of 78 records were initially identified through a comprehensive electronic database search (Web of Science *n* = 12; EBSCO *n* = 16; Scopus *n* = 15; EMBASE *n* = 35). After the removal of duplicates (*n* = 40), 38 records were screened by title and abstract together, of which 17 were excluded against the eligibility criteria. The remaining 21 full-text articles were assessed for eligibility. Of these, 16 were excluded (systematic reviews *n* = 3; narrative reviews *n* = 3; sample size < 3 patients *n* = 8; descriptive case series without outcome data *n* = 1; treatment protocol without outcome data *n* = 1). Consequently, five studies were included in the review (Figure 1). Inter-rater agreement at the full-text eligibility stage was good (κ = 0.70; Pr(a) = 0.905; Pr(e) = 0.687). Outcome measurements across the included studies relied predominantly on two-dimensional radiography. Malmgren et al. used standardised periapical and panoramic radiographs with the three-point Malmgren scoring system; Zhang et al. used periapical radiographs and clinical examination to quantify infraocclusion; Lin et al. used standardised periapical radiographs and clinical calliper measurements of bucco-palatal ridge width; Kaán et al. used periapical radiographs supplemented by cone-beam computed tomography (CBCT) in selected cases; and Han et al. combined periapical and panoramic radiographs with clinical photographic documentation. CBCT was not employed systematically, which limited accurate three-dimensional assessment of volumetric ridge change across the evidence base.

### 5.2. Characteristics of the Included Studies

The five included studies comprised two retrospective cohort studies [8,17] and three case series [18,19,20]. Publications ranged from 2013 to 2025 and examined a total of 140 decoronated permanent teeth with follow-up periods from 1 to 30 years. Sample sizes ranged from three to 103 patients and collectively included 140 decoronated teeth. Across the included studies, the affected teeth were predominantly previously erupted permanent anterior teeth, mainly incisors, that developed ankylosis and progressive IO following traumatic injuries such as avulsion or luxation. The characteristics of the included studies are outlined in Table 4.

### 5.3. Vertical Bone Preservation

Four of the five included studies reported on vertical alveolar bone level (ABL) changes using the Malmgren three-point scoring system (Score 1, unchanged or reduced ABL; Score 2, moderate increase; Score 3, considerable increase). Malmgren et al. [8] reported that patients treated at around 14.6 years (boys) and 13.0 years (girls) achieved Score 3, whereas post-pubertal patients (boys = 16.8 years; girls = 17.3 years) mainly exhibited Score 1 results. In addition, Zhang et al. [17] corroborated this pattern, reporting 11 of 12 decoronated teeth achieved Score 3, with optimal outcomes at 11.25 ± 1.77 years (girls) and 12.12 ± 0.83 years (boys). However, Kaán et al. [19] reported more heterogeneous outcomes, partly attributable to the inclusion of post-skeletally mature patients and cases of external cervical rather than replacement resorption.

### 5.4. Horizontal Bone Changes

The studies that measured ridge width reported a persistent horizontal bone deficiency post-decoronation. For instance, Lin et al. [18] (the only study with quantitative data) reported a statistically significant decrease in bucco-palatal ridge width at the decoronated site (9 ± 1.0 mm) compared with the contralateral tooth (10.17 ± 0.9 mm), with a mean loss of 1.67 ± 1.12 mm (*p* = 0.004). Furthermore, the horizontal ridge reduced over time (*p* = 0.027). The case series by Han et al. [20] also described the same pattern, with vertical ABL dimensions maintained but horizontal reductions observed across all three cases.

### 5.5. Infraocclusion

Decoronation was consistently linked to stabilisation or improvement of IO. Zhang et al. [17] reported IO reductions of approximately 2.2 mm in boys and 3.2 mm in girls and identified a relationship between root development stage and severity of infraposition (teeth at earlier stages showed worse IO). Furthermore, Han et al. [20] demonstrated IO improvements across all three cases during long-term follow-up of up to 10 years. Lastly, Malmgren et al. [8] also observed reduced IO discrepancy among younger patients undergoing prepubertal intervention.

### 5.6. Timing of Decoronation

Across the included studies, the timing of decoronation emerged as one of the key factors to influence treatment outcomes. Both Malmgren et al. [8] and Zhang et al. [17] (*p* < 0.05) reported that Score 3 outcomes were associated with early decoronation, particularly when performed before 13–14 years in girls and 14–15 years in boys. In contrast, post-pubertal treatment mainly yielded Score 1 outcomes. Furthermore, decoronation performed in girls around 2 years earlier compared to boys exhibited more favourable ridge preservation, which aligns with the earlier onset of skeletal maturation in females [8,17,19].

### 5.7. Methodological Quality and Risk of Bias

The included studies demonstrated moderate to good methodological quality, as shown in Table 5. Malmgren et al. [8] and Zhang et al. [17] scored 7/9 on NOS (low risk of bias). Among the case series, Lin et al. [18] scored 7/10, Kaán et al. [19] scored 9/10 (both low risk), and Han et al. [20] scored 6/10 (moderate risk) owing to the limited sample size and incomplete reporting of eligibility criteria. The main methodological limitations across studies were the absence of a comparator group of non-decoronated teeth, reliance on two-dimensional radiographic measurement, and heterogeneous outcome definitions.

**Figure 2:**
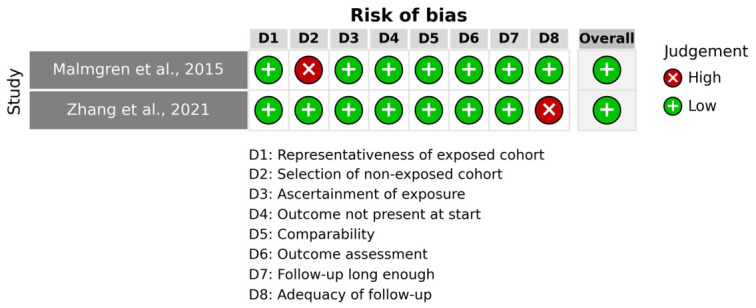
Risk of bias assessment of cohort studies using the Newcastle–Ottawa Scale [15]. Malmgren et al., 2015 [8], Zhang et al., 2021 [17].

**Figure 3:**
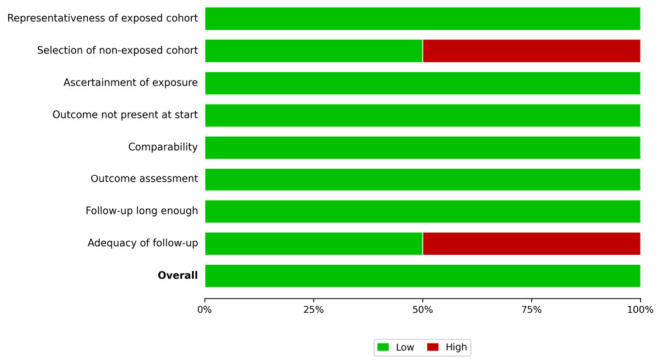
Risk of bias summary across domains of the Newcastle–Ottawa Scale for included cohort studies [15].

**Figure 4:**
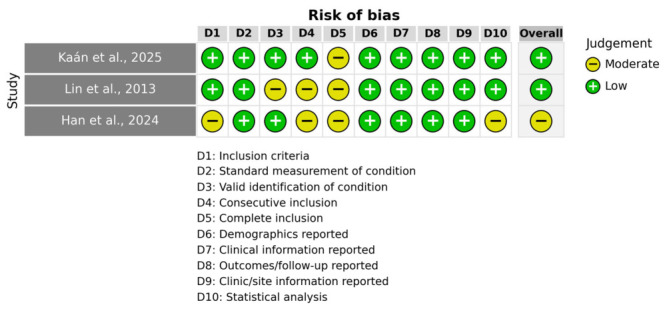
Risk of bias assessment of case series using the Joanna Briggs Institute Critical Appraisal Checklist [16]. Kaán et al., 2025 [19], Lin et al., 2013 [18], Han et al., 2024 [20].

**Figure 5:**
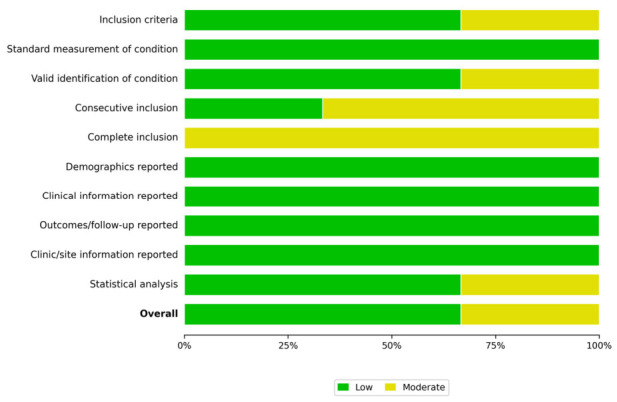
Risk of bias summary across domains of the JBI Critical Appraisal Checklist for included case series [16].

Because the included studies employed different designs, the risk of bias was visualised separately by appraisal tool. Cohort studies were assessed using NOS (Figure 2 and Figure 3), and case series were assessed using the JBI Critical Appraisal Checklist (Figure 4 and Figure 5).

## 6. Discussion

This review synthesises the current clinical evidence on decoronation as a ridge-preservation strategy for DA in this population. Following the comprehensive database search, only five studies met the eligibility criteria, comprising two cohort studies and three case series. A total of 140 decoronated teeth were assessed across the following domains: vertical and horizontal bone changes, IO progression and future implant site feasibility.

A direct comparison between decoronation and extraction was not possible because none of the included studies used a controlled extraction comparator. Therefore, the question of whether decoronation preserves ridge dimensions better than extraction cannot be answered directly and must be interpreted using indirect post-extraction ridge-resorption evidence, which is discussed in detail below.

Despite consistent findings on vertical bone preservation, the limited number of studies and their observational design significantly restrict the strength of conclusions. The absence of controlled comparisons with extraction represents a major gap in the literature and limits the ability to establish decoronation as a superior intervention. From a clinical perspective, decoronation may be considered a preferred conservative option in growing patients with ankylosed anterior permanent teeth when preservation of the alveolar ridge for future implant rehabilitation is a priority, provided that treatment timing, growth stage, and patient-specific factors are carefully evaluated.

### 6.1. Vertical Bone Preservation and the Biological Rationale of Decoronation

The most consistent finding of this review is that prepubertal decoronation is associated with preservation or enhancement of vertical ABL in this population. As reported by both cohort studies [8,17], Score 3 ABL outcomes were observed in early intervention, whilst delayed treatment exhibited Score 1 levels. This finding was supported by the biological rationale described by Malmgren et al. [11], where the root left in situ served as a matrix for bone apposition during active craniofacial growth. Simultaneously, the severed interdental fibres reorganise between neighbouring teeth, contributing to bone apposition through the dentoperiosteal fibre complex. However, this regenerative mechanism declines upon completion of skeletal maturity as bone apposition diminishes [11]. Therefore, individual growth stages are an essential factor in treatment success rather than chronological age.

### 6.2. Horizontal Ridge Reduction

Despite the generally positive outcome for bone height growth, a reduction in alveolar width was consistently reported across the studies. In particular, the case series by Lin et al. (2013) quantified a progressive decrease in bucco-palatal ridge width over time, with a mean loss of 1.67 ± 1.12 mm compared to the contralateral healthy tooth [18]. These findings were corroborated by Han et al. (2024) [20]. The resorption is predominantly labial because the two cortical plates are made of different bone. The buccal cortex of the anterior maxilla is largely bundle bone—PDL-dependent tissue that loses its biological maintenance signal once replacement resorption destroys the PDL. The palatal cortex, composed mainly of lamellar bone, is more resistant. Therefore, when labial bone loss is more pronounced, guided bone regeneration or other ridge augmentation procedures may be needed at the time of implant placement [20].

### 6.3. Comparison with Extraction—Indirect Evidence

No study in the current literature directly compared decoronation with extraction in the same patient population. Indirect comparison with post-extraction ridge-resorption data is therefore necessary. Schropp et al. [21] reported that alveolar ridge width decreased by approximately 50% within one-year post-extraction, with two-thirds of this loss occurring within the first three months. Tan et al. [22] reported an average horizontal bone loss of 3.79 ± 0.23 mm and vertical loss of 1.24 ± 0.11 mm at six months after extraction. These values are substantially higher than the 1.67 mm of horizontal loss Lin et al. [18] recorded after decoronation. These findings suggest that decoronation may better preserve ridge width than exodontia, despite the indirect nature of this comparison. Nonetheless, this interpretation must be taken with caution, as the extraction studies included non-ankylosed teeth in predominantly adult populations.

Extraction of ankylosed teeth also carries additional risks compared with standard extraction. In particular, extraction in cases with extensive replacement resorption on the buccal plate may result in avulsion of the attached alveolar bone, producing combined vertical and horizontal defects that may not heal spontaneously [9]. This is particularly concerning in the anterior maxillary region of these individuals, where the thin buccal cortex is most vulnerable.

### 6.4. Infraocclusion and Timing of Intervention

All studies reporting this outcome found that IO stabilised after decoronation. Zhang et al. [17] also showed that teeth at earlier stages of root formation (stages 2 and 3) exhibited severe IO as opposed to those nearing complete development (stages 4 and 5). That said, this reinforces the argument for earlier intervention in younger patients. Furthermore, early work by Malmgren and Malmgren [23] recommended that decoronation be performed within 2–3 years of diagnosis in children under 10 years of age to prevent severe IO and bone defects, and highlighted that vertical growers exhibit worse IO progression than horizontal growers.

The findings point toward early intervention in these patients with evident or anticipated IO progression. Additionally, timing should be individualised based on growth pattern and pubertal stage.

### 6.5. Implications for Implant Rehabilitation

None of the included studies reported direct implant placement outcomes, despite this being arguably the most clinically relevant indicator of successful ridge preservation. For instance, Malmgren et al. [8] reported that 14 of 18 individuals who later received implants required no ridge augmentation. This finding, albeit indirect evidence, still suggests that decoronation can preserve sufficient bone volume for implant rehabilitation.

Davarpanah et al. [24] further challenged the traditional requirement for implants to be placed in native bone, reporting favourable osseointegration when implants were placed directly into retained ankylosed root fragments over a 42-month follow-up. However, implant survival data after decoronation remain scarce, representing the most important evidence gap identified by the present review.

Broader comparative evidence from adjacent fields reinforces this biological rationale. In adult implant dentistry, the intentional retention of root structure to preserve the surrounding bone underpins partial extraction therapy and, in particular, the socket-shield technique, in which a buccal root fragment is deliberately left in situ at implant placement to maintain the buccal bundle bone and the overlying soft-tissue contour [25,26]. As with decoronation, this approach relies on the periodontal-ligament-dependent vascular supply of the retained fragment to limit buccal plate resorption, and reported outcomes show reduced ridge collapse relative to conventional extraction. The convergence of these independent lines of evidence—paediatric decoronation and adult partial extraction therapy—strengthens the broader conclusion that preserving, rather than removing, root structure better maintains alveolar architecture. Equally, the recognised complications of partial extraction therapy, such as fragment migration and incomplete healing, indicate that meticulous case selection and surgical technique remain decisive, a caveat that applies to decoronation in growing patients as well.

### 6.6. Comparison with Previous Evidence

The first systematic review on this topic, by Mohadeb et al. [13] in 2016, included 12 heterogeneous reports published between 1984 and 2015 and reported a mean horizontal ridge loss of 1.67 mm following decoronation—a finding consistent with the quantitative data included in the present review. That review, however, did not apply a minimum follow-up threshold, pooled short- and long-term data, and has since been overtaken by nearly a decade of new clinical evidence. More recently, Bautista et al. [27] published a systematic review in March 2025 that examined the role of decoronation in vertical bone preservation and growth. Their review adds useful synthesis on the vertical dimension, and identifies some overlap with the clinical questions addressed here.

The present review differs from Bautista et al. in four deliberate respects. For instance, their synthesis centred on vertical bone changes, whereas this study additionally examined horizontal ridge modification, IO progression, pubertal stage as a modifying factor, and implications for future implant placement. These outcomes collectively aimed to determine whether decoronation functions as a viable rehabilitative alternative procedure.

In addition, the established eligibility criteria in this study extended to retrospective cohort studies, not only case series. This enabled the inclusion of Malmgren et al. [8] (*n* = 103), the largest clinical dataset available on this topic and one that case-series-only criteria would have excluded. This review also compared decoronation outcomes against post-extraction resorption data [21,22], a clinical reference point that, to our knowledge, no prior synthesis has included.

Lastly, we assessed risk of bias with design-matched tools—NOS for cohort studies, JBI for case series—rather than applying a single instrument regardless of study design. The two reviews are not redundant: Bautista et al. address vertical bone change; this review covers wider outcomes to inform clinical decision-making in these patients.

### 6.7. Limitations

This systematic review was unable to draw definitive conclusions due to the small number of included studies (*n* = 5), the nature of the study designs, the lack of randomised controlled trials, and the heterogeneity in outcome assessment methods. All these collectively precluded the synthesis of a meta-analysis. Furthermore, the comparison between decoronation and extraction was evaluated based on indirect evidence due to the absence of comparative studies in the current literature. Additionally, there was substantial heterogeneity in outcome measurements. For instance, only Zhang et al. [17] quantified IO progression, whereas Lin et al. [18] conducted the only study to measure horizontal bone changes. The remaining included studies used 2D periapical radiographs that failed to capture bucco-palatal dimensions. Finally, none of the included studies reported direct implant placement outcomes. The overall level of evidence remains low, highlighting the need for well-designed, prospective, controlled clinical studies. In addition, the deliberately specific search strategy and stringent eligibility criteria, while ensuring direct relevance to the clinical question, are inherent constraints that contributed to the small number of studies identified. The updated search (May 2026) confirmed that no further eligible studies have been published.

### 6.8. Clinical Implications

Despite these limitations, decoronation may be considered a clinically relevant option in growing patients with ankylosed anterior teeth, particularly when preservation of vertical alveolar bone is a priority. The procedure appears most beneficial when performed during active growth, before significant infraocclusion develops. Optimal outcomes depend on coordinated multidisciplinary management. The paediatric or general dentist typically establishes the initial diagnosis and refers the patient; the orthodontist manages space and occlusal relationships during growth; the oral surgeon performs the decoronation and any subsequent bone augmentation; and the prosthodontist or implantologist plans and delivers definitive rehabilitation once growth is complete. This interdisciplinary coordination, from diagnosis through to final implant rehabilitation, is essential to optimise functional and prosthetic outcomes.

However, clinicians should be aware that horizontal ridge reduction is likely to occur and may necessitate future augmentation procedures. Treatment planning should therefore incorporate a long-term perspective, balancing immediate biological preservation with eventual prosthetic requirements. Patient selection, growth assessment, and careful timing remain critical to optimising outcomes.

#### Clinical Decision Framework

When to consider decoronation:

- Prepubertal patients.
- Ankylosed anterior teeth.
- High aesthetic demand.

When caution is needed:

- Late diagnosis.
- Severe buccal bone loss.
- Poor compliance.

### 6.9. Future Research Directions

Future research should prioritise well-designed prospective cohort studies with standardised protocols and, where feasible, controlled comparisons with extraction. The use of three-dimensional imaging modalities, such as CBCT, is essential for accurate assessment of volumetric bone changes. Standardised outcome measures—including quantitative ridge dimensions, infraocclusion indices, and implant success rates—should be adopted to improve comparability.

Long-term studies evaluating implant placement following decoronation are particularly needed to establish definitive clinical guidelines. Additionally, integration of growth assessment tools would allow more precise evaluation of the relationship between treatment timing and outcomes.

#### Overall Interpretation

In summary, decoronation represents a biologically sound and clinically promising approach for managing ankylosed teeth in growing patients. The current evidence supports its role in preserving vertical alveolar bone and stabilising infraocclusion; however, consistent horizontal ridge reduction and the absence of high-quality comparative and implant outcome data limit the strength of recommendations. Until more robust evidence becomes available, decoronation should be considered within an individualised, multidisciplinary treatment framework.

## 7. Conclusions

This systematic review suggests that decoronation is a clinically promising treatment for preserving vertical alveolar bone in growing patients with ankylosed teeth, particularly when performed before or during the pubertal growth phase. The procedure also appears to stabilise infraocclusion, supporting favourable functional and aesthetic outcomes.

However, consistent horizontal bone reduction was observed, and evidence regarding long-term implant conditions remains limited. Although indirect comparisons suggest that decoronation may better preserve ridge dimensions than extraction, the lack of controlled comparative studies precludes definitive conclusions.

Overall, decoronation represents a promising, biologically based treatment strategy in this population. Nevertheless, the current evidence is limited by small sample sizes, observational study designs, and heterogeneity in outcome assessment. Further high-quality prospective studies are required to establish standardised clinical protocols and confirm long-term outcomes.

## Figures and Tables

**Table 1:** Inclusion criteria.

Category	Inclusion Criteria
Study design	Clinical trials, retrospective or prospective cohort studies, case–control studies, or case series with a sample size ≥ 3 patients.
Patient population	Human studies involving growing patients (prepubertal, pubertal, or adolescent) with ankylosed permanent anterior teeth; ankylosis associated with replacement resorption; mean patient age ≤ 18 years if growth stage was not stated.
Intervention	Studies evaluating decoronation as a treatment modality; studies comparing decoronation with extraction; minimum follow-up ≥6 months.
Comparator	Extraction; natural progression without intervention (if ridge outcomes were assessed).
Outcome variables	Alveolar bone height or width changes, ridge maintenance, infraocclusion progression, implant feasibility or placement, or aesthetic outcomes with a clear measurement method.
Publication criteria	Publications in English or Spanish (January 2006 to May 2026); peer-reviewed full-text articles.

**Table 2:** Exclusion criteria.

Category	Exclusion Criteria
Study design	Animal studies; in vitro studies; biomechanical simulations; systematic reviews; meta-analyses; expert opinions; case reports ≤ 3 patients; narrative reviews.
Patient population	Primary teeth; non-ankylosed cases; patients after growth completion; posterior teeth; studies including mixed adult/paediatric samples without separate data.
Intervention	Studies focused solely on inflammatory root resorption; studies evaluating other interventions without decoronation for DA treatment.
Outcome variables	Studies not reporting bone preservation or clinical growth outcomes; histological outcomes without clinical relevance; follow-up ≤6 months; unclear diagnostic criteria for DA.
Publication characteristics	Articles without full-text availability or not peer-reviewed.

**Table 3:** Summary of database-specific search concepts and Boolean operators.

Database	Keywords & Boolean Operators
EBSCO/MEDLINE	(tooth ankylosis OR ankylosed tooth OR dental ankylosis) AND (adolescents OR pubertal) AND (alveolar bone loss OR alveolar bone preservation) AND (decoronation OR coronectomy OR surgical crown removal)
Web of Science	(ankylosed OR ankylosis OR replacement resorption) AND (decoronation OR crown amputation OR decrowning OR coronectomy) AND (permanent tooth) AND (growing OR adolescent OR mixed dentition OR pubertal) AND (alveolar bone OR bone height OR bone width OR infraocclusion OR ridge preservation)
Scopus	Ankylosis AND young AND decoronation AND (alveolar ridge OR alveolar bone)
EMBASE	(tooth ankylosis OR ankylosed OR replacement resorption) AND (decoronation OR crown amputation OR coronectomy) AND (permanent tooth OR incisor) AND (adolescent OR growing OR mixed dentition) AND (alveolar ridge OR bone height OR bone width OR infraocclusion)

**Table 4:** Summary of study characteristics and main outcomes of included studies on decoronation in ankylosed permanent anterior teeth in growing patients.

Author, Year	Study Design	Sample Size	Follow-Up	Main Findings
Malmgren et al., 2015 [8]	Retrospective longitudinal cohort	103	1–30 years	Score 3 vertical alveolar bone-level outcome reported in younger patients; poorer outcomes reported after pubertal growth phase.
Zhang et al., 2021 [17]	Retrospective cohort	12	3–10 years	11/12 teeth achieved Score 3; IO reduced by 2.2 mm in boys and 3.2 mm in girls; best outcomes at 11–12 years of age.
Kaán et al., 2025 [19]	Retrospective case series	9	1.5–8.5 years	Heterogeneous outcomes; favourable bone gain only in prepubertal subgroup; confounded by post-skeletal-maturity cases.
* Lin et al., 2013 [18]	Comparative case series	12	1–6.8 years	Quantitative bucco-palatal ridge loss of 1.67 ± 1.12 mm vs. contralateral tooth (*p* = 0.004); ridge width decreased over time.
Han et al., 2024 [20]	Case series	4 (3 patients)	3–10 years	Vertical ABL maintained; consistent horizontal bone reduction; IO stabilisation across all cases.

*: Only study with quantitative horizontal ridge-width data; vertical bone height was not measured. Abbreviations: IO, infraocclusion; ABL, alveolar bone level.

**Table 5:** Methodological quality assessment and overall risk of bias of the included studies.

Study	Design	Tool	Score	Overall RoB	Main Limitation
Malmgren et al., 2015 [8]	Retrospective cohort	NOS	7/9	Low	No true non-exposed cohort.
Zhang et al., 2021 [17]	Retrospective cohort	NOS	7/9	Low	Follow-up completeness declined over time.
Kaán et al., 2025 [19]	Retrospective case series	JBI	9/10	Low	Complete inclusion of eligible cases not fully confirmed.
Lin et al., 2013 [18]	Comparative case series	JBI	7/10	Low	Unclear consecutive and complete inclusion.
Han et al., 2024 [20]	Case series	JBI	6/10	Moderate	Very small sample and limited selection detail.

## Data Availability

No new data were created or analysed in this study. Data sharing is not applicable to this article.

## Supplementary Materials

The following supporting information can be downloaded at: https://www.mdpi.com/article/10.3390/healthcare14131811/s1, Table S1: PRISMA 2020 checklist; Table S2: GRADE Summary of Findings; Table S3: Full-text articles excluded, with reasons (*n* = 16). Reference [28] is cited in the supplementary materials.
