# Supplementary material for: Decoronation as a Surgical Technique for Managing Ankylosed Permanent Anterior Teeth in Growing Patients: A Systematic Review
Source: Healthcare (Basel). 2026 Jun 23;14(13):1811. doi: 10.3390/healthcare14131811 (PMC13361940; doi:10.3390/healthcare14131811)
Supplement: Supplementary file 1 [file healthcare-14-01811-s001.zip › healthcare-4348891-supplementary.pdf]

Supplementary Materials

Decoronation as a Surgical Technique for Managing Ankylosed Permanent Anterior Teeth in Growing Patients: A Systematic Review

Table S1. PRISMA 2020 checklist.

Section references correspond to the revised manuscript headings; page numbers should be confirmed against the final typeset layout. From: Page MJ, McKenzie JE, Bossuyt PM, et al. The PRISMA 2020 statement: an updated guideline for reporting systematic reviews. BMJ. 2021;372:n71. doi:10.1136/bmj.n71.

Section and Topic	Item #	Checklist item	Location where item is reported
**TITLE**			
	**1**	Identify the report as a systematic review.	Title
**ABSTRACT**			
	**2**	See the PRISMA 2020 for Abstracts checklist.	Abstract
**INTRODUCTION**			
	**3**	Describe the rationale for the review in the context of existing knowledge.	Section 1 (Introduction)
	**4**	Provide an explicit statement of the objective(s) or question(s) the review addresses.	Section 2 (Research Question); Section 3 (Objectives)
**METHODS**			
	**5**	Specify the inclusion and exclusion criteria for the review and how studies were grouped for the syntheses.	Section 4.2; Tables 1 & 2
	**6**	Specify all databases, registers, websites, organizations, reference lists and other sources searched or consulted to identify studies. Specify the date when each source was last searched or consulted.	Section 4.3
	**7**	Present the full search strategies for all databases, registers and websites, including any filters and limits used.	Section 4.3; Table 3
	**8**	Specify the methods used to decide whether a study met the inclusion criteria of the review, including how many reviewers screened each record and each report retrieved, whether they worked independently, and if applicable, details of automation tools used in the process.	Section 4.3
	**9**	Specify the methods used to collect data from reports, including how many reviewers collected data from each report, whether they worked independently, any processes for obtaining or confirming data from study investigators, and if applicable, details of automation tools used in the process.	Section 4.4
	**10a**	List and define all outcomes for which data were sought. Specify whether all results that were compatible with each outcome domain in each study were sought, and if not, the methods used to decide which results to collect.	Section 4.1 (PICO); Section 4.4
	**10b**	List and define all other variables for which data were sought. Describe any assumptions made about any missing or unclear information.	Section 4.4
	**11**	Specify the methods used to assess risk of bias in the included studies, including details of the tool(s) used, how many reviewers assessed each study and whether they worked independently, and if applicable, details of automation tools used in the process.	Section 4.5
	**12**	Specify for each outcome the effect measure(s) (e.g. risk ratio, mean difference) used in the synthesis or presentation of results.	Section 4.4; Section 5 (narrative)
	**13a**	Describe the processes used to decide which studies were eligible for each synthesis.	Section 4.4
	**13b**	Describe any methods required to prepare the data for presentation or synthesis, such as handling of missing summary statistics, or data conversions.	Section 4.4
	**13c**	Describe any methods used to tabulate or visually display results of individual studies and syntheses.	Section 4.4; Table 4

Section and Topic	Item #	Checklist item	Location where item is reported
	**13d**	Describe any methods used to synthesize results and provide a rationale for the choice(s). If meta-analysis was performed, describe the model(s), method(s) to identify the presence and extent of statistical heterogeneity, and software package(s) used.	Section 4.4 (narrative synthesis; no meta-analysis performed)
	**13e**	Describe any methods used to explore possible causes of heterogeneity among study results.	Not applicable (no meta-analysis)
	**13f**	Describe any sensitivity analyses conducted to assess robustness of the synthesized results.	Not applicable (no meta-analysis)
	**14**	Describe any methods used to assess risk of bias due to missing results in a synthesis (arising from reporting biases).	Not applicable (narrative synthesis)
	**15**	Describe any methods used to assess certainty (or confidence) in the body of evidence for an outcome.	Section 4.5 (GRADE)
**RESULTS**			
	**16a**	Describe the results of the search and selection process, from the number of records identified in the search to the number of studies included in the review, ideally using a flow diagram.	Section 5.1; Figure 1 (PRISMA flow diagram)
	**16b**	Cite studies that might appear to meet the inclusion criteria, but which were excluded, and explain why they were excluded.	Section 5.1; Supplementary Table S3
	**17**	Cite each included study and present its characteristics.	Section 5.2; Table 4
	**18**	Present assessments of risk of bias for each included study.	Section 5.7; Table 5; Figures 2–5
	**19**	For all outcomes, present, for each study: summary statistics for each group (where appropriate) and an effect estimate and its precision, ideally using structured tables or plots.	Sections 5.3–5.6; Table 4
	**20a**	For each synthesis, briefly summarize the characteristics and risk of bias among contributing studies.	Sections 5.2–5.7
	**20b**	Present results of all statistical syntheses conducted. If meta-analysis was done, present for each the summary estimate and its precision and measures of statistical heterogeneity.	Sections 5.3–5.6 (narrative; no meta-analysis)
	**20c**	Present results of all investigations of possible causes of heterogeneity among study results.	Not applicable (no meta-analysis)
	**20d**	Present results of all sensitivity analyses conducted to assess the robustness of the synthesized results.	Not applicable (no meta-analysis)
	**21**	Present assessments of risk of bias due to missing results (arising from reporting biases) for each synthesis assessed.	Not applicable (narrative synthesis)
	**22**	Present assessments of certainty (or confidence) in the body of evidence for each outcome assessed.	Section 5.7; Supplementary Table S2 (GRADE)
**DISCUSSION**			
	**23a**	Provide a general interpretation of the results in the context of other evidence.	Section 6 (Discussion)
	**23b**	Discuss any limitations of the evidence included in the review.	Section 6.7
	**23c**	Discuss any limitations of the review processes used.	Section 6.7
	**23d**	Discuss implications of the results for practice, policy, and future research.	Sections 6.8 & 6.9
**OTHER INFORMATION**			
	**24a**	Provide registration information for the review, including register name and registration number, or state that the review was not registered.	Section 4.1 (OSF: https://osf.io/kbn8j)
	**24b**	Indicate where the review protocol can be accessed, or state that a protocol was not prepared.	Section 4.1 (OSF registration)
	**24c**	Describe and explain any amendments to information provided at registration or in the protocol.	Not applicable (no amendments)

Section and Topic	Item #	Checklist item	Location where item is reported
	**25**	Describe sources of financial or non-financial support for the review, and the role of the funders or sponsors in the review.	Funding statement
	**26**	Declare any competing interests of review authors.	Conflicts of Interest statement
	**27**	Report which of the following are publicly available and where they can be found: template data collection forms; data extracted from included studies; data used for all analyses; analytic code; any other materials used in the review.	Data Availability Statement

Table S2. GRADE summary of findings.

The certainty of evidence for each outcome was appraised using the Grading of Recommendations, Assessment, Development and Evaluations (GRADE) framework. As all included studies were observational in design (retrospective cohorts and case series), the starting certainty for each outcome was set at LOW. Evidence was then assessed across five domains — risk of bias, inconsistency, indirectness, imprecision, and other considerations (including dose-response and large effect) — and downgraded or upgraded accordingly. Final ratings are interpreted as: ⊕⊕⊕⊕ **High**, ⊕⊕⊕○ **Moderate**, ⊕⊕○○ **Low**, ⊕○○○ **Very low**.

Outcome	Nº of studies (n)	Study design	Risk of bias	Inconsistency	Indirectness	Imprecision	Findings	Certainty of evidence
**Vertical alveolar bone preservation**	4 studies (n = 127 patients)	2 retrospective cohort studies + 2 case series	Not serious	Not serious	Not serious	Serious ([a])	Pre-pubertal decoronation was consistently associated with vertical alveolar bone gain (Score 3 on the Malmgren scale) across both cohort studies. Post-pubertal cases predominantly exhibited Score 1 outcomes (unchanged or reduced bone level). Outcomes were upgraded once for a clear age-dependent dose-response gradient observed across the two cohort studies.[b]	⊕⊕○○ **LOW**
**Horizontal ridge changes (bucco-palatal width)**	2 studies (n = 15 teeth)	2 case series	Not serious	Not serious	Not serious	Very serious ([c])	Persistent horizontal ridge reduction was reported across all included cases. Lin et al. (n = 12) reported a mean bucco-palatal loss of 1.67 ± 1.12 mm relative to the contralateral tooth ($p = 0.004$), with progressive reduction over time ($p = 0.027$). Han et al. described qualitative horizontal loss in all 3 cases.	⊕○○○ **VERY LOW**
**Infraocclusion progression**	3 studies (n = 118 patients)	2 retrospective cohort studies + 1 case series	Not serious	Not serious	Not serious	Serious ([a])	Decoronation was consistently associated with stabilisation or improvement of infraocclusion. Zhang et al. quantified IO reductions of approximately 2.2 mm in boys and 3.2 mm in girls; Han et al. and Malmgren et al. described concordant qualitative improvements across follow-up periods of up to 30 years.	⊕○○○ **VERY LOW**
**Effect of intervention timing (growth stage)**	2 studies (n = 115 patients)	2 retrospective cohort studies	Not serious	Not serious	Not serious	Not serious	Both cohort studies showed a statistically significant association between earlier intervention and	⊕⊕○○ **LOW**

Outcome	Nº of studies (n)	Study design	Risk of bias	Inconsistency	Indirectness	Imprecision	Findings	Certainty of evidence
							improved bone outcomes ($p < 0.05$ in both). Score 3 outcomes were obtained when decoronation was performed before age 14.6 years (boys) and 13.0 years (girls). The earlier optimal age in girls is consistent with their earlier skeletal maturation.	
**Implant site feasibility**	0 studies	—	—	—	—	—	No included study evaluated implant placement as a primary outcome. This represents a critical evidence gap, as implant rehabilitation is one of the principal long-term clinical objectives of decoronation in growing patients. Direct evidence is required from prospective studies with implant outcomes.	**—**<br>**Evidence unavailable**
**Aesthetic outcomes (gingival contour and soft-tissue profile)**	2 studies	1 retrospective cohort + 1 case series	Not serious	Not serious	Serious ([d])	Serious ([a])	Favourable preservation of the gingival contour was reported following decoronation. However, progressive horizontal ridge reduction may compromise the long-term soft-tissue profile, and aesthetic outcomes were not assessed using standardised or validated tools.	⨁◯◯◯<br>**VERY LOW**

Footnotes:

[a] Downgraded once for serious imprecision: limited number of studies and small total sample size, with no meta-analytic estimate or pooled confidence intervals available.

[b] Upgraded once for a clear dose-response gradient: across both cohort studies, earlier intervention age was independently and consistently associated with greater vertical bone preservation (Malmgren et al. [8], $p < 0.05$; Zhang et al. [17], $p < 0.05$).

[c] Downgraded twice for very serious imprecision: only one study (Lin et al., $n = 12$) reported quantitative bucco-palatal measurements; remaining evidence was qualitative.

[d] Downgraded once for indirectness: aesthetic outcomes were not assessed using standardised or validated instruments and were reported only narratively.

Reference: Guyatt GH, Oxman AD, Vist GE, Kunz R, Falck-Ytter Y, Alonso-Coello P, et al. GRADE: an emerging consensus on rating quality of evidence and strength of recommendations. *BMJ*. 2008;336(7650):924–926. doi:10.1136/bmj.39489.470347.AD.

Table S3. Full-text articles excluded, with reasons (n = 16).

Of the 21 full-text articles assessed for eligibility, 16 were excluded for the reasons listed below.

No.	Excluded study	Database	Reason for exclusion
**1**	Cohenca N, Stabholz A. Decoronation — a conservative method to treat ankylosed teeth for preservation of alveolar ridge prior to permanent prosthetic reconstruction: literature review and case presentation. Dent Traumatol. 2007;23(2):87–94.	EBSCO	Sample size < 3 patients
**2**	Jaikaria A, Thakur S. Alveolar ridge preservation in a growing patient with decoronation: one-year follow-up. J Indian Soc Pedod Prev Dent. 2019;37(2):214–217.	EBSCO	Sample size < 3 patients
**3**	Sapir S, Shapira J. Decoronation for the management of an ankylosed young permanent tooth. Dent Traumatol. 2008;24(1):131–5.	Web of Science	Sample size < 3 patients
**4**	Son WS, Kim SS, Kim SR, Kwon JW. Management of an ankylosed incisor occurring during adolescence using alveolar bone distraction osteogenesis and decoronation: case report. J Dent Rehabil Appl Sci. 2017;33(2):143–151.	Web of Science	Sample size < 3 patients
**5**	Lima TFR, Soares AJ, Zaia AA. Multidisciplinary approach for replacement root resorption following severe intrusive luxation: a case report of decoronation. 2017.	EMBASE	Sample size < 3 patients
**6**	Shay B, Mijiritsky E, Bronstein M, Ben Simhon T. Flapless decoronation: a minimally invasive approach. Int J Environ Res Public Health. 2023;20(1):603.	EMBASE	Sample size < 3 patients
**7**	Sapir S, Kalter A, Sapir MR. Decoronation of an ankylosed permanent incisor: alveolar ridge preservation and rehabilitation by an implant-supported porcelain crown. 2009.	Scopus	Sample size < 3 patients
**8**	Kadian B, Kadian S, Sharma A. Management of a delayed reimplanted avulsed young permanent tooth by decoronation: a case report. 2021.	Scopus	Sample size < 3 patients
**9**	Mohadeb JVN, Somar M, He H. Effectiveness of decoronation technique in the treatment of ankylosis: a systematic review. Dent Traumatol. 2016;32(4):255–63.	EBSCO	Systematic review
**10**	Bautista A, Ghilotti J, Sanz JL, Llena C. Decoronation as a therapeutic alternative for ankylosis in children and adolescents for vertical bone preservation and growth: a systematic review. J Clin Med. 2025;14(6):1945.	EBSCO	Systematic review
**11**	Einy S, Kridin K, Kaufman AY, Cohenca N. Immediate post-operative rehabilitation after decoronation: a systematic review. Dent Traumatol. 2020;36(2):141–150.	EMBASE	Systematic review
**12**	Malmgren B. Ridge preservation/decoronation. J Endod. 2013;39(3 Suppl): S67–72.	EBSCO	Narrative review
**13**	Kosem R. Decoronation in treating ankylosis. 2018.	EMBASE	Narrative review
**14**	Sigurdsson A. Decoronation as an approach to treat ankylosis in growing children. Pediatr Dent. 2009;31(2):123–128.	Scopus	Narrative review
**15**	Siddiqui MM, Patel M, Shahdad S. Spontaneous alveolar bone growth in ankylosed, infraoccluded teeth in adolescents after elective decoronation — a clinical case series. Dent Update. 2016;43(3):206–210.	EBSCO	Descriptive case series without outcome data (decoronation not assessed as the primary outcome)
**16**	Lin S, Fuss Z, Wigler R, Karawani M, Ashkenazi M. Decoronation: treatment protocol for ankylotic root resorption as a consequence of dental trauma. 2013.	Scopus	Treatment protocol without outcome data
